# Five-Year Comparative Efficacy of Everolimus-Eluting vs. Resolute Zotarolimus-Eluting Stents in Patients with Acute Coronary Syndrome Undergoing Percutaneous Coronary Intervention

**DOI:** 10.3390/jcm10061278

**Published:** 2021-03-19

**Authors:** Endrin Koni, Wojciech Wanha, Jakub Ratajczak, Zhongheng Zhang, Przemysław Podhajski, Rita L. Musci, Giuseppe M. Sangiorgi, Maciej Kaźmierski, Antonio Buffon, Jacek Kubica, Wojciech Wojakowski, Eliano P. Navarese

**Affiliations:** 1 Department of Interventional Cardiology, Santa Corona Hospital, 17027 Pietra Ligure, Italy; endrinkoni@gmail.com; 2 SIRIO MEDICINE Research Network, 85094 Bydgoszcz, Poland; 3 Department of Cardiology and Structural Heart Diseases, Medical University of Silesia, 40635 Katowice, Poland; wojciech.wanha@gmail.com (W.W.); kazmierski.maciej@gmail.com (M.K.); wojtek.wojakowski@gmail.com (W.W.); 4 Department of Cardiology and Internal Medicine, Nicolaus Copernicus University, 87100 Bydgoszcz, Poland; ratajczak.j.m@gmail.com (J.R.); przemo.podhajski@gmail.com (P.P.); jwkubica@gmail.com (J.K.); 5 Department of Health Promotion, Nicolaus Copernicus University, 87100 Bydgoszcz, Poland; 6 Department of Emergency Medicine, Sir Run-Run Shaw Hospital, Zhejiang University School of Medicine, Hangzhou 310009, China; zh_zhang1984@zju.edu.cn; 7 Key Laboratory of Emergency and Trauma, Ministry of Education, College of Emergency and Trauma, Hainan Medical University, Haikou 571199, China; 8 Department of Biomedicine and Prevention, University of Rome Tor Vergata, 00173 Rome, Italy; muscir45@gmail.com; 9 Cardiac Cath Lab, Department of Cardiology, San Gaudenzio Clinic, 28100 Novara, Italy; gsangiorgi@gmail.com; 10 Institute of Cardiology, Catholic University of the Sacred Heart Rome, 00168 Rome, Italy; antonino.buffon@unicatt.it; 11 Faculty of Medicine, University of Alberta, Edmonton, AB 13103, Canada

**Keywords:** everolimus-eluting stent, resolute zotarolimus-eluting stent, acute coronary syndrome

## Abstract

Among drug-eluting stents (DESs), the durable polymer everolimus-eluting stent (EES) and resolute zotarolimus-eluting stent (R-ZES) are widely used in clinical practice and have contributed to improve the outcomes of patients undergoing percutaneous coronary intervention (PCI). Few studies addressed their long-term comparative performance in patients with acute coronary syndrome (ACS). We aimed to investigate the 5 year comparative efficacy of EES and R-ZES in ACS. We queried ACTION-ACS, a large-scale database of ACS patients undergoing PCI. The treatment groups were analyzed using propensity score matching. The primary endpoint was a composite of mortality, myocardial infarction (MI), stroke, repeat PCI, and definite or probable stent thrombosis, which was addressed at the five-year follow-up. A total of 3497 matched patients were analyzed. Compared with R-ZES, a significant reduction in the primary endpoint at 5 years was observed in patients treated with EES (hazard ratio (HR) [95%CI] = 0.62 [0.54–0.71], *p* < 0.001). By landmark analysis, differences between the two devices emerged after the first year and were maintained thereafter. The individual endpoints of mortality (HR [95%CI] = 0.70 [0.58–0.84], *p* < 0.01), MI (HR [95%CI] = 0.55 [0.42–0.74], *p* < 0.001), and repeat PCI (HR [95%CI] = 0.65 [0.53–0.73], *p* < 0.001) were all significantly lower in the EES-treated patients. Stroke risk did not differ between EES and R-ZES. In ACS, a greater long-term clinical efficacy with EES vs. R-ZES was observed. This difference became significant after the first year of the ACS episode and persisted thereafter.

## 1. Introduction

The advent of modern, second-generation coronary drug-eluting stents (DESs) has contributed to improve the outcomes of patients with coronary artery disease (CAD) undergoing percutaneous coronary intervention (PCI) by refining metallic design, polymer coatings, and the introduction of newer antiproliferative drugs [1]. Among CAD patients, those with acute coronary syndrome (ACS) constitute a high-risk cohort experiencing more frequent adverse clinical events [2]. Extensive research in ACS has mainly been conducted on outcomes at the short or medium-term follow-up [3]. However, there is a paucity of data concerning the long-term comparison of second-generation DES in the ACS context.

Zotarolimus-eluting resolute stents (R-ZESs) (Medtronic) and everolimus-eluting stents (EESs), such as Xience (Abbott Vascular) and Promus Element (Boston Scientific), are second-generation DES widely used in clinical practice. Two studies have reported comparable outcomes in the long-term between these devices [4,5]. However, they were all-comers trials with many stable CAD patients included.

Within this framework, a relevant clinical question is whether DES-type selection in ACS may affect long-term outcomes in these patients. We aimed to investigate the 5-year efficacy profile of these durable polymer DES devices (R-ZES vs. EES) in ACS patients undergoing PCI, by querying a large-scale multicenter prospective registry.

## 2. Methods

**Figure 1:**
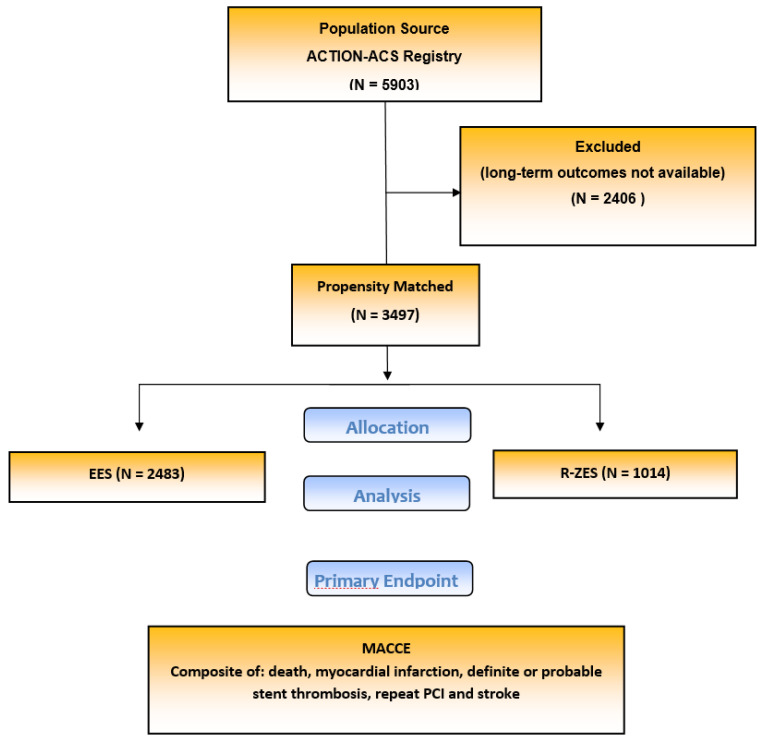
Study flow chart. EES, everolimus-eluting stent; R-ZES, resolute zotarolimus-eluting stent; MACCE, major adverse cardiovascular and cerebrovascular events; PCI, percutaneous coronary intervention.

We retrospectively analyzed the ACTION-ACS, a pooled large patient ACS database involving two interventional large-volume academic centers based in Poland: the Department of Cardiology and Structural Heart Diseases, Medical University of Silesia, Katowice, and the Nicolaus Copernicus University, Bydgoszcz, Poland. Information on the follow-up events was site-reported and adjudicated by a trained physician-investigator. Data of consecutive patients with ACS, undergoing PCI with EES (Xience, Abbott Vascular, Santa Clara, CA, USA and Promus Element, Boston Scientific, Natick, MA, USA) or R-ZES (Resolute Integrity, Resolute Onyx, Medtronic, Fridley, MN, USA), were collected between November 2009 and February 2017 (Figure 1). Relevant baseline information, procedural, and clinical outcomes at follow-up were entered into prespecified electronic case report forms. Angioplasty and stent selection were performed according to standard techniques at the discretion of the interventional cardiologist. All patients were prescribed dual antiplatelet therapy (DAPT) consisting of acetylsalicylic acid, 75 to 100 mg daily, and a P2Y12 inhibitor for at least 1 year according to guidelines.

### 2.1. Study End Points

The primary prespecified endpoint, major adverse cardiovascular and cerebrovascular events (MACCEs), defined as a composite of death, myocardial infarction (MI), definite or probable stent thrombosis, repeat PCI and stroke, was addressed at the five year follow-up [6].

Th secondary endpoints included death, repeat PCI, MI, and stroke. MI was defined according to its fourth universal definition [7].

### 2.2. Statistical Analysis

Categorical variables are reported as n (%) and continuous variables as means ± standard deviation. Categorical variables were compared by χ^2^ or Fisher’s exact tests, as appropriate. Continuous data were analyzed by the independent-samples *t*-test.

We performed an adjusted analysis based on the propensity score (PS). The PS is the probability that each individual patient is included in the treatment group and was estimated via logistic regression based on the available baseline covariates [8]. Potential confounders were entered into the PS model based on known clinical relevance of associations (*p* < 0.01) observed at univariate analysis. The final variable selection was performed by a logistic or Cox regression model with least absolute shrinkage and selection operator (LASSO) penalty and a tuning parameter selected by cross-validation, which allows minimizing overfitting [9].

Missing data were present in less than 5% of inspected variables. Assuming that data were missing at random, we used polytomous logistic regression, logistic regression, and predictive mean matching as multiple imputation techniques to fill in missing values, using the R mice package. Matching was performed with the use of a full matching algorithm that minimizes biases [10]. The covariate balance was assessed by exploring the standardized mean differences between unadjusted and adjusted populations and distribution of the PS. The standardized differences were estimated for all the baseline covariates before and after matching to assess the pre-match imbalance and the post-match balance. Standardized differences of less than 10.0% for a given covariate indicate a relatively small imbalance.

The Cox proportional-hazards regression analyses were performed on the matched pairs. The results of the Cox regression at the 5 year follow-up is presented both as an unadjusted and adjusted hazard ratio (HR) with a 95% confidence interval (CI). Kaplan–Meier curves were generated for the endpoints of interest and a landmark survival analysis was conducted, setting one year as the landmark time. A *p* value < 0.05 was considered statistically significant for all analyses. For the subgroup analyses, the p interaction was calculated and a value of <0.10 was considered significant. The adjusted statistical analysis was performed using R 4.0 with mice, survival and matchthem packages.

## 3. Results

From an initial cohort of 5903 unmatched patients, a total of 3497 matched patients with ACS were analyzed. The remainder was excluded because long-term outcomes were not complete. All ACS patients with non-ST segment elevation myocardial infarction (NSTEMI) or segment elevation myocardial infarction (STEMI) had acute myocardial injury with clinical evidence of acute myocardial ischemia according to the fourth universal definition of myocardial infarction. Among the propensity-matched patients, 3007 (86%) were NSTEMI/ unstable angina (UA) and 490 (14%) STEMI. The clinical characteristics in the EES and R-ZES groups are illustrated in Table 1. The average age was 65.1 years in the EES group vs. 64.9 years in the R-ZES group. Women represented 32.3% and 33.5%, respectively. The diabetic patients represented 34.7% and 35.7%, respectively. A radial access was predominant in both groups (88% vs. 90.6%). Multivessel disease was present in less than 20% of the treated groups. Angina class III–IV was observed in 73.2% and 75.4% of the treated patients, respectively. There was no acute stent thrombosis in either group. The mean follow-up was 1686 days in the EES group and 1652 days in the R-ZES group.

### 3.1. Primary Endpoint

**Figure 2:**
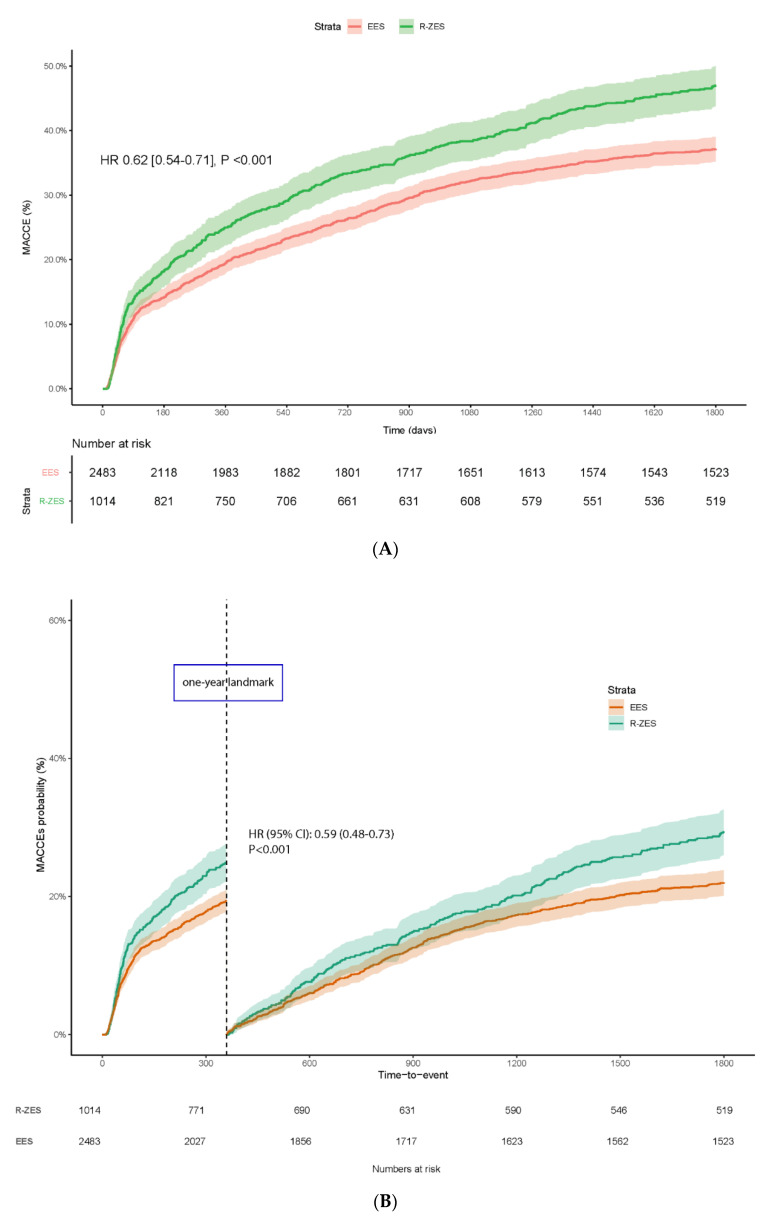
(**A**) Kaplan–Meier graph of the cumulative incidence of MACCE. (**B**) The time-to-event landmark analysis showing event curve divergence that became statistically significant after the one year landmark point.

Patients treated with EES stent had a significantly lower risk of MACCE at 5 years: 36.8% vs. 46.3% (HR [95% CI] = 0.62 [0.54–0.71], *p* < 0.001, Figure 2A). By landmark analysis, the cumulative rate difference between the two devices became statistically significant after one year (Figure 2B). Although the separation of the curves was observed earlier, within the first year, there was no significant difference between EES and R-ZES: HR [95% CI] = 0.98 [0.81–1.19], *p* = 0.88. In contrast, a significant MACCE reduction emerged after the first year of follow-up: HR [95%CI] = 0.59 [0.48–0.73], *p* < 0.001.

### 3.2. Secondary Endpoints

#### All-Cause Mortality

**Figure 3:**
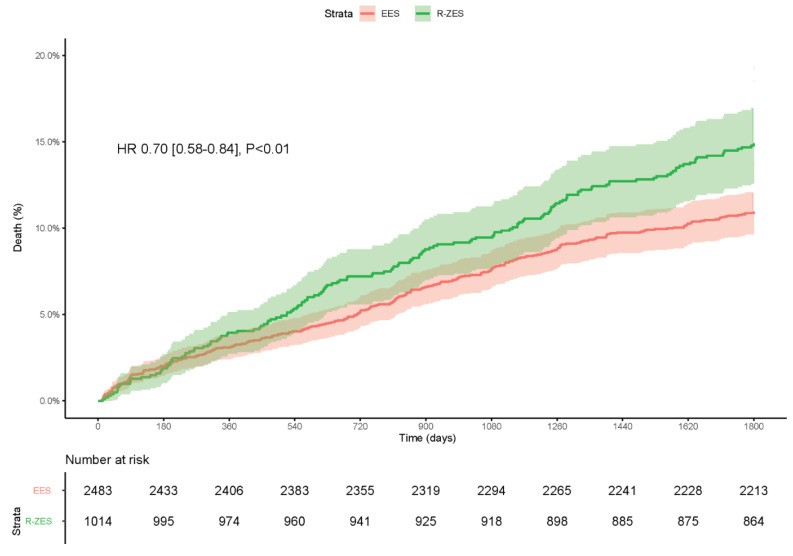
Kaplan–Meier graph of the cumulative incidence of mortality.

Patients receiving a EES had significantly lower all-cause mortality rates compared with those treated with a R-ZES: 12.6% vs. 17.6%, HR [95% CI] = 0.70 [0.58–0.84], *p* < 0.01 (Figure 3).

### 3.3. Myocardial Infarction

**Figure 4:**
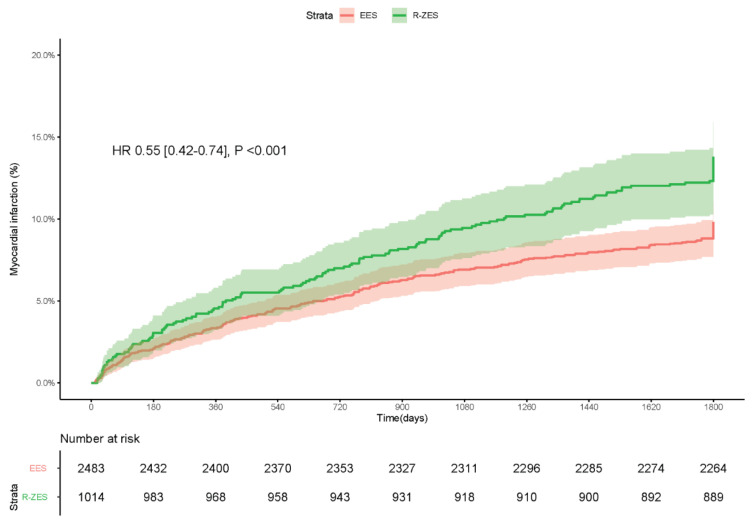
Kaplan–Meier graph of the cumulative incidence of myocardial infarction.

The DES-treated patients allocated to the EES cohort experienced significantly lower rates of myocardial infarction compared with those treated with a R-ZES: 9.8% vs. 13.8%, HR [95% CI] = 0.55 [0.42–0.74], *p* < 0.001 (Figure 4).

### 3.4. Repeat PCI and Stroke

**Figure 5:**
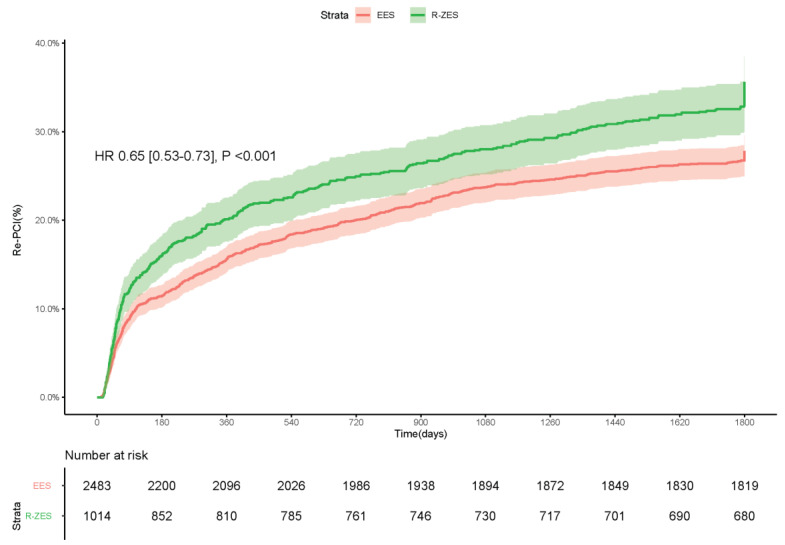
Kaplan–Meier graph of the cumulative incidence of repeat PCI.

A significantly lower number of repeat PCI procedures were observed in patients treated with EES vs. R-ZES: 27.8% vs. 35.6%, HR [95%CI] = 0.65 [0.53–0.73], *p* < 0.001 (Figure 5).

No significant difference was found in stroke risk between EES and R-ZES: 4.7% vs. 5.4%: HR [95%CI] = 0.84 [0.61–1.17], *p* = 0.32.

### 3.5. DES Performance in Pre-Specified Subgroups

**Figure 6:**
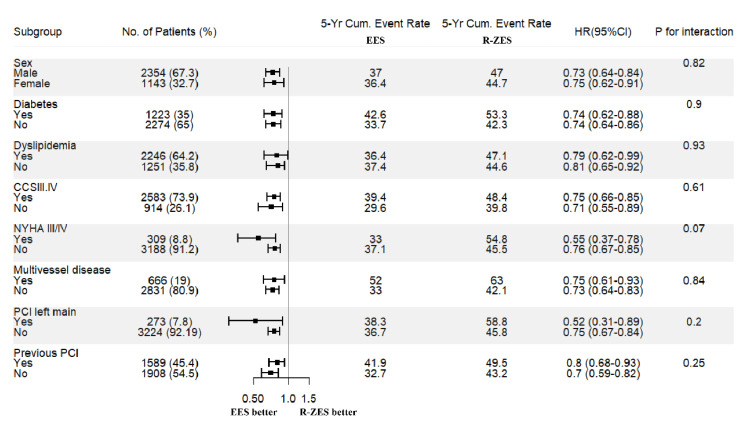
Forest plot analysis of the prespecified subgroups. CCS, Canadian Cardiovascular Society Grading Angina; NYHA, New York Heart Association heart failure classification; PCI, percutaneous coronary intervention.

Several prespecified subgroups were explored to compare EES with R-ZES. They included sex, diabetes, dyslipidemia, Canadian Cardiovascular Society (CCS) III–IV class angina, New York Heart Association (NYHA) III–IV, multivessel disease, left main PCI, and previous PCI. The estimates were directionally consistent in favor of EES in magnitude and direction without significant interactions (Figure 6).

## 4. Discussion

The main findings of this large-scale analysis of 3497 ACS propensity-matched patients treated with EES or R-ZES drug-eluting stents followed up to five years are the following: (1) compared with R-ZES, EES yielded a reduced risk of the primary composite endpoint of mortality, myocardial infarction, stroke, repeated percutaneous coronary intervention, and stent thrombosis; (2) mortality was significantly reduced in the EES-treated patients; (3) a significant reduction in individual ischemic endpoints of MI and repeat PCI was observed; (4) by landmark survival analysis, we identified the temporal window of greater event reduction with EES, which occurred after one year, while no significant differences between the EES and R-ZES emerged earlier; (5) the reduction in adverse events in the EES cohort was consistent in all prespecified subgroups.

Newer-generation permanent polymer DESs offer numerous improvements over their first-generation counterparts [11]. Each of the three stent components (metallic backbone, polymer coating, and antiproliferative drug) has undergone refinements. These features include decreased strut thickness, improved flexibility/deliverability, enhanced polymer biocompatibility/drug elution profiles, and superior re-endothelialization kinetics [12,13]. Available platforms are composed of cobalt-chrome or platinum-chrome. Cobalt alloys are widely used in new-generation DESs as they provide better radiopacity and radial strength [14].

Currently, ACS remains a high-risk setting associated with worse outcomes than stable patients [15,16,17]. Within this framework, many factors can affect the long-term results of DES and one of those is the polymer, which has been linked to an enhanced vascular inflammatory response [18]. The enduring inflammatory response might lead to late stent thrombosis and restenosis owing to a combination of delayed re-endothelialization, late-acquired incomplete apposition, neointimal hyperplasia, and neoatherosclerosis, which in turn can impact long-term clinical outcomes [19,20,21,22,23].

In the ACS setting, vascular healing is delayed at the culprit site of the DES implantation for an ACS episode compared with stable CAD [24]. One of the most relevant complications with the use of first-generation DES was the high incidence of late and very late stent thrombosis (ST) concerning a bare metal stent (BMS) [24,25,26]. Although the advent of second-generation DESs afterward has reduced the incidence of ST, their permanent metallic scaffold prevents a complete recovery of vascular structure and function, which may have implications on very late stent failure [1,27]. In this regard, DES components, such as the drug-eluting polymer and their biocompatibility, are key determinants for long-term device success. ACS is associated with higher risk of adverse outcomes owing to the proinflammatory state and higher atherosclerotic burden [16]. Besides pharmacological and technology advancement, a persistent risk of adverse events remains over the 1 year period after an ACS event [15,28,29]. Thus, a DES design aimed to provide a more biocompatible polymer and a better polymer/drug elution balance remains a pivotal goal in ACS [21,30].

In both EES and R-ZES platforms, drug release is completed within 180 days [31]. Thus, it is unlikely that the observed long-term stent differences between these two DES in our study were drug-related. Conversely, the associated durable polymer could play a key role in the modulation of DES performance over the long-term. Accordingly, R-ZESs incorporate BioLinx, which is a mixture of hydrophobic C10, hydrophilic C19, and polyvinyl pyrrolidone polymers. Polymer-orientation results in a hydrophilic surface and hydrophobic core with enhanced biocompatibility [32]. At variance with R-ZESs, the EES polymer consists of a bilayer copolymer (vinylidene fluoride-co-hexafluoropropylene) (PVDF-HFP), which is highly fluorinated and referred to as a fluoropolymer [33]. The two components of the polymer demonstrate high biocompatibility. Fluorinated copolymers, such as vinylidene fluoride–hexafluoropropylene copolymer, reduce platelet adhesion and thrombus formation [34]. These features are likely related to a high retention of albumin, which in turn passivates the stent surface and prevents fibrinogen binding [35]. Fluoropolymer coated with everolimus stent platforms also induces a lower inflammatory response, even compared with a BMS [36].

These polymer characteristics could be implicated in greater long-term ischemia reduction and may offer improved survival in patients treated with an EES platform compared with R-ZES, as noted in our study. Although no substantial differences between these two devices has emerged in a stable setting, our findings of long-term clinical improvement with EES vs. R-ZES in ACS are in agreement with other studies that demonstrated an incremental benefit with new-generation DES in relation to ACS severity [37].

The R-ZES and EES are the most-used durable polymer platforms among DES. The RESOLUTE US, a prospective observational study of resolute [38] and SPIRIT FIRST [39], a trial comparing Xience with a bare metal stent, demonstrated a good long-term outcome for both devices. Subsequently, four studies conducted a short-term (1 year) head-to-head comparison of R-ZES with EES [40,41,42,43]. They enrolled mainly stable CAD patients with only one of them including STEMI patients. Thus, a paucity of comparative data between EES and R-ZES is available in the ACS field. The resolute all-comers trial compared long-term results of R-ZES with Xience EES [44]. Among the included patients in the trial, only 34% had an MI. Resolute and Xience stents yielded a similar efficacy and safety. In the subgroup analysis of patients with an acute MI, which included 662 patients only, there was a numerical nonsignificant benefit in favor of EES. The DUTCH PEERS randomized study evaluated the 5 year outcomes of Resolute Integrity ZES and Promus Element EES [4]. Approximately 42% of included patients were stable CAD. Resolute Integrity and Promus Element showed similar efficacy. However, in this study, the comparative performance between devices was not addressed in the pure ACS setting.

These studies were underpowered to conclude on individual endpoints in ACS patients.

One study evaluated very late (1–5 years) pathological response to EES (EES; Abbott Vascular) and BMS (Multilink Vision; Abbott Vascular). A lower inflammatory response of the EES was observed concerning the BMS platform [45]. The pathology findings translated into better clinical outcomes in the EXAMINATION trial that included 1498 patients with STEMI [46]. At 1 year, the composite endpoint (all-cause death, MI, or any revascularization) did not differ between the two groups (11.9% vs. 14.2%, *p* = 0.19). Notably, at the 5 year follow-up, there were significantly lower rates of the composite endpoint in the EES treatment group compared with the BMS (21% vs. 26%, *p* = 0.03).

To the best of our knowledge, this is one of the largest-scale analyses of propensity-matched patients at long-term after ACS. We observed at 5-year follow-up greater clinical benefits with EES in comparison to R-ZES. This difference became significant after the first year of the index event and persisted thereafter. Further randomized and adequately powered research is warranted to confirm the relative long-term efficacy of EES vs. R-ZES in these high-risk patients.

## 5. Limitations

This was an observational study and, unlike a randomized trial, does not account for all confounding variables. The use of a propensity score generated a balanced population, mitigating confounding. However, residual confounding could not be fully excluded. Consistency of the results in all prespecified subgroups supports the reliability of the findings. Propensity matching generated balanced groups in terms of their baseline characteristics. Less complete information was available with precise timing at the follow-up for stent thrombosis, which made it impossible to explore it as an individual endpoint. Most patients included in the registry with available 5 year data were NSTEMI or UA, while a lower fraction had a STEMI. We did not include ACS type in the propensity matching procedure since UA and NSTEMI are part of the continuum of ACS, which also includes STEMI. All ACS patients with NSTEMI or STEMI had acute myocardial injury with clinical evidence of acute myocardial ischemia according to the fourth universal definition of MI. The inclusion of ACS type in the propensity analysis would have led to a greater loss of statical power by reducing the final sample size. In some instances, multiple stents were implanted; however, they belonged to the same class, thereby limiting potential confounding arising from different stents.

Because a significant difference between the two devices was observed beyond the first year only, it is unlikely that any periprocedural factor occurring during the invasive procedure between treatment groups could have influenced long-term outcomes. However, given the observational design, these findings of greater efficacy of EES vs. R-ZES need further validation in future powered randomized trials.

## 6. Conclusions

In this large-scale analysis performed in ACS patients, treatment with EES resulted in better long-term outcomes compared with R-ZES. The benefit was observed after the first year and persisted thereafter. These findings suggest that in patients with ACS, EESs provide a greater 5-year clinical efficacy in comparison with R-ZESs. Further adequately powered investigations are needed to address the long-term efficacy of these devices in ACS.

## Figures and Tables

**Table 1:** The baseline characteristics after propensity matching of ACS patients treated with EES vs. R-ZES.

Patient Characteristics	*EES*	*R-ZES*	*p* Value
	(n = 2483)	(n = 1014)	
Women, n (%)	803 (32.3)	340 (33.5)	0.7
Age, mean ± SD	65.16 (10.3)	64.99 (10.2)	0.4
Diabetes, n (%)	861 (34.7)	362 (35.7)	0.5
CCSIII.IV, n (%)	1818 (73.2)	765 (75.4)	0.1
Hypertension, n (%)	2131 (85.8)	881 (86.9)	0.4
BMI, n (%)	28.44 (4.4)	28.16 (4.1)	0.2
Dyslipidemia, n (%)	1575 (63.4)	671 (66.2)	0.1
Anemia, n (%)	388 (15.6)	153 (15.1)	0.6
NYHA, n (%)			0.3
NYHA class II	295 (11.9)	100 (9.9)	
NYHA class III	174 (7)	64 (6.3)	
NYHA class IV	51 (2.1)	20 (2.0)	
Radial access, n (%)	2185 (88.0)	919 (90.6)	0.3
MVD, n (%)	474 (19.1)	192 (18.9)	0.9
Bifurcation, n (%)	92 (3.7)	40 (3.9)	0.7
**Adjunctive therapies**			
IABP, n (%)	25 (1.0)	9 (0.9)	0.7
Thrombectomy, n (%)	52 (2.0)	19 (1.8)	0.2
GP IIB/IIIa inh, n(%)	151 (6.1)	52 (5.1)	0.4
**Culprit vessel PCI**			
PCI Cx, n (%)	596 (24.0)	224 (22.1)	0.2
PCI LAD, n (%)	1135 (45.7)	469 (46.3)	0.7
PCI RCA, n (%)	654 (26.3)	258 (25.4)	0.5
PCI LM, n (%)	107 (4.3)	34 (3.4)	0.1
Residual stenosis, n (%)	13 (0.5)	4 (0.4)	0.4

SD = standard deviation; BMI = body mass index; LAD = left anterior descending; Cx = circumflex artery; GP IIB/IIIa inh = glycoprotein IIb/IIIa inhibitor; LM = left main; RCA = right coronary artery; IABP = intraortic balloon pump counterpulsation; MVD = multivessel disease; NYHA = New York Heart Association heart failure classification; CCS = Canadian Cardiovascular Society Grading Angina.

## Data Availability

Upon reasonable request after all authors’ signed approval.

## Abbreviations

ACS	acute coronary syndrome
ACTION-ACS	international acute coronary syndrome registry
BMS	bare metal stents
CAD	coronary artery disease
CCS	Canadian Cardiovascular Society Grading Angina
DES	drug-eluting stents
EES	everolimus-eluting stents
MACCE	major adverse cardiovascular and cerebrovascular events
MI	myocardial infarction
NSTEMI	Non-ST-Segment Elevation Myocardial Infarction
NYHA	New York Heart Association heart failure classification
PCI	percutaneous coronary intervention
R-ZES	Resolute zotarolimus-eluting stents
STEMI-ST	Segment Elevation Myocardial Infarction
UA	unstable angina
